# Adefovir dipivoxil-induced hypophosphatemic osteomalacia and osteoporosis: a case report and literature review

**DOI:** 10.3389/fendo.2026.1766477

**Published:** 2026-03-17

**Authors:** Wenshu Yu, Chunxia Deng, Shangyu Chen, Hui Jiang, Jiaqin Jiang

**Affiliations:** 1 Department of Endocrinology, The People’s Hospital of Guangxi Zhuang Autonomous Region, Nanning, China; 2 Department of Pharmacy, The People’s Hospital of Guangxi Zhuang Autonomous Region, Nanning, China

**Keywords:** adefovir dipivoxil, Fanconi syndrome, hypophosphatemic osteomalacia, osteoporosis, pseudofracture

## Abstract

The nephrotoxicity of adefovir dipivoxil (ADV) can induce Fanconi syndrome, which causes hypophosphatemic osteomalacia. We report a case of a 67-year-old postmenopausal woman who had been receiving long-term adefovir dipivoxil therapy for chronic hepatitis B. She presented with severe bone pain and multiple pseudofractures, and was diagnosed with drug-induced hypophosphatemic osteomalacia. Following discontinuation of adefovir and supplementation with vitamin D and phosphate, her biochemical parameters normalized and bone pain significantly improved, indicating that osteomalacia had been essentially corrected. However, although follow-up bone mineral density (BMD) showed significant improvement compared to pretreatment values, it remained markedly below the reference range for age-matched women. Postmenopausal osteoporosis was therefore suspected, and anti-osteoporotic pharmacotherapy was initiated after correction of osteomalacia. This case suggests that BMD can improve substantially after removal of the causative factor in drug-induced osteomalacia; however, if patients have underlying risk factors for osteoporosis, low BMD may persist even after osteomalacia resolution, necessitating further evaluation and intervention.

## 1 Introduction

Chronic HBV is a viral infection disease with a high prevalence rate in China. Clinically, patients are advised to undergo long-term antiviral treatment regimens (1). Among current first-line agents, ADV is widely used. As a nucleotide analogue, it is effective against both wild-type HBV and lamivudine-resistant strains (2). However, with prolonged treatment, some patients may develop adverse effects in the renal and skeletal systems, manifesting as decreased bone mineral density, diminished muscle strength, and increased fracture risk, ultimately compromising overall mobility and quality of life (3) [3].

Recent years have seen a growing number of reports of ADV–induced hypophosphatemic osteomalacia and Fanconi syndrome (4), because its clinical picture mimics osteoporosis, hypophosphatemic osteomalacia is frequently misdiagnosed (5). We report a case of adefovir dipivoxil-associated hypophosphatemic osteomalacia. Following discontinuation of adefovir and correction of metabolic abnormalities, bone mineral density (BMD) improved significantly but remained below the reference range for age-matched peers, suggesting possible concomitant postmenopausal osteoporosis. This case highlights that in postmenopausal women receiving long-term adefovir dipivoxil therapy who present with severe bone pain and low BMD, osteomalacia should be identified and managed first. After metabolic parameters stabilize, further evaluation for primary osteoporosis should be conducted to avoid missed diagnosis or overdiagnosis.

## 2 Case report

In December 2022, a 67-year-old woman presented with generalized polyarthralgia that was exacerbated by walking and persisted at rest. She did not seek formal medical care at the onset of these symptoms. Between November and December 2023, she noted a marked escalation of generalized joint pain—most intense in the bilateral ribs, lumbosacral region, and hips—rendering her unable to walk or find comfort in either sitting or lying down. On December 26, 2023, she was admitted to our orthopedic service via wheelchair. She had no prior history of fracture. Her medical history was significant for a diagnosis of HBV carriage at an outside hospital in 2016, with subsequent continuous antiviral therapy using ADV. She underwent menopause at age 49 and had a gynecological history of G2P2 (two gravidities, two parities). She denied any other significant personal or family history of genetic disorders.

Physical examination revealed a temperature of 36.4 °C, heart rate 72 beats/min, respiratory rate 20 breaths/min, blood pressure 115/62 mmHg, height 138 cm, weight 41 kg, and BMI 21.5 kg/m². Physical examination revealed a positive thoracic compression test, a notable kyphotic deformity of the spine, and increased thoracic kyphosis beyond the normal physiologic range; no focal tenderness, percussion pain, or paraspinal tenderness was elicited.

**Figure 1:**
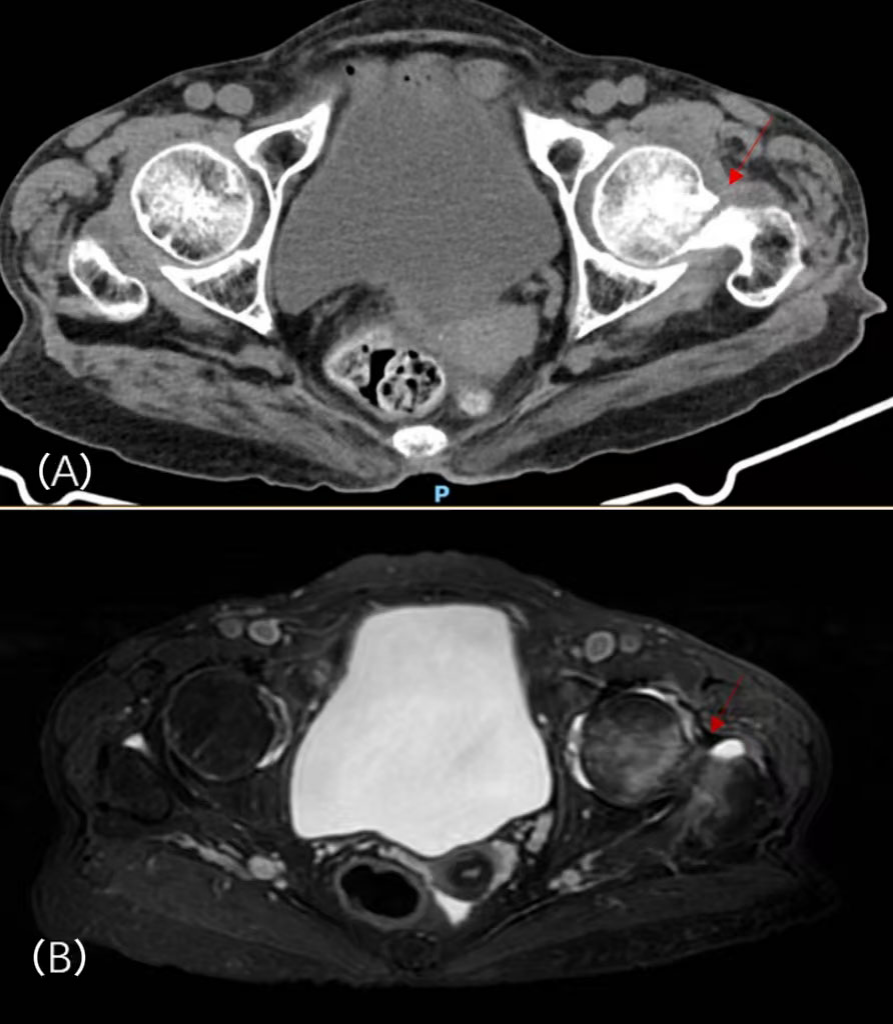
Hip CT **(A)** and hip MRI **(B)** before treatment.

**Figure 2:**
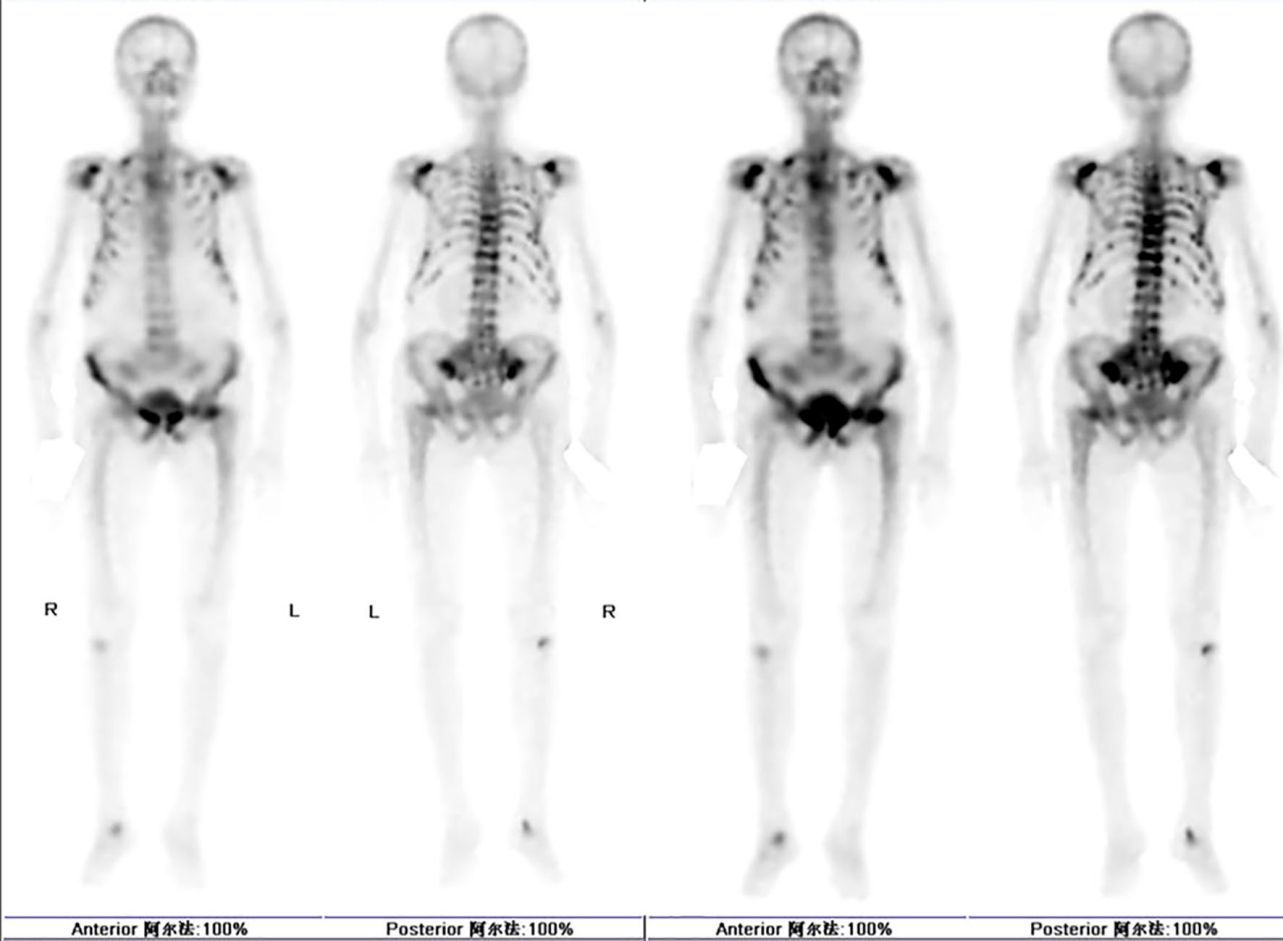
Emission computed tomograph (ECT) whole-body bone imaging.

Laboratory data disclosed hypophosphatemia, hyperchloremia, total hypocalcemia, hypokalemia, and an elevated alkaline phosphatase 1. Lumbar-spine MRI: compression deformities of L1 and L3 vertebrae, with mild compression changes at L2, L4, and L5. Hip CT raised concern for a left femoral-neck fracture; hip MRI suggested an impacted left femoral-neck fracture with associated bone-marrow edema and bilateral ischiofemoral impingement syndrome (**Figure 1**). Dual-energy X-ray absorptiometry (DXA) showed low bone density with T-scores of -5.3 for the total lumbar spine, -4.1 for the left femoral neck, and -4.6 for the total left hip2. Additionally, whole-body bone scintigraphy (ECT) indicated a high probability of multiple fractures throughout the skeleton (**Figure 2**). The Orthopedics department initiated management with calcium and vitamin D supplementation, tramadol for analgesia, and denosumab 60 mg for osteoporosis. However, her generalized bone pain persisted despite this treatment, prompting her transfer to our department on January 5, 2024.

Further workup in our department revealed renal tubular acidosis, elevated bone turnover markers, and an elevated fractional excretion of phosphate (FEP). Furthermore, hematologic, rheumatologic, and oncologic disorders—such as multiple myeloma, rheumatoid arthritis, and other cancerous bone metastases—were systematically excluded (**Table 1**). In view of her seven-year history of ADV therapy for HBV, we reached a working diagnosis of hypophosphatemic osteomalacia complicating Fanconi syndrome.

**Table 1:** Patient’s laboratory findings.

Examination	Normal range	Jan.2024	Aug.2024	Oct.2024	Feb.2025	Aug.2
Serum						
PH	7.35-7.45	7.29	–	–	7.43	–
K(mmol/L)	3.5-5.3	3.49	–	4.24	3.68	3.74
Na(mmol/L)	137-147	140	–	142	141	141
Cl(mmol/L)	99-110	116	–	106	109	109
Total Ca(mmol/L)	2.11-2.52	1.89	–	2.25	2.15	2.22
P(mmol/L)	0.85-1.51	0.37	–	0.94	1.04	1.12
Mg(mmol/L)	0.75-1.02	0.9	–	1.04	0.88	1.03
Cr(ummol/L)	45-84	69.8	–	–	96	86
ALP(U/L)	50-135	424	300	200	101	74
B-ALP (ug/L)	≤ 22.4	–	106.6	68.15	16.23	–
HCO (mmol/L)	22-27	14.9	–	–	20.6	–
PTH (pg/mL)	*12-88	26.52	–	40.84	30.47	32.66
25(OH)D(ng/mL)*	<20:Deficient	–	54.16	–	30.94	–
25(OH)D(nmol/L)**	<75:Deficient	62.36	–	–	–	143
-CTX (ng/mL)	<1.008	0.78	4.38	0.1	0.32	0.78
PINP (ng/mL)	20.25-76.31	258	466	134	59.1	48.6
Urine						
Protein	Negative	2+	–	–	Negative	–
Total urine phosphorus in 24h(mmol/24h)	12.9-42	11.56	–	–	10.85	13.78
FEP (%)		46.9	–	–	–	–

K, Potassium; Na, Sodium; Cl, Chlorine; Total Ca, Total Calcium; P, Phosphorus; Mg, Magnesium; Cr, Creatinine; ALP, Alkaline phosphatase; B-ALP, Bone alkaline phosphatase; HCO_3_^-^,Bicarbonate (from blood gas analysis);PTH, Parathyroid Hormone; 25(OH)D, 25-Hydroxyvitamin D; β-CTX, C-terminal telopeptide of type I collagen; PINP, Procollagen Type I Intact N-Terminal Propeptide; FEP, fractional excretion of phosphate, The formula for calculation is FEP = (urinary phosphorus × serum creatinine)/(serum phosphorus × urinary creatinine) × 100%; * Based on mass spectrometry. **Based on chemiluminescence.

**Figure 3:**
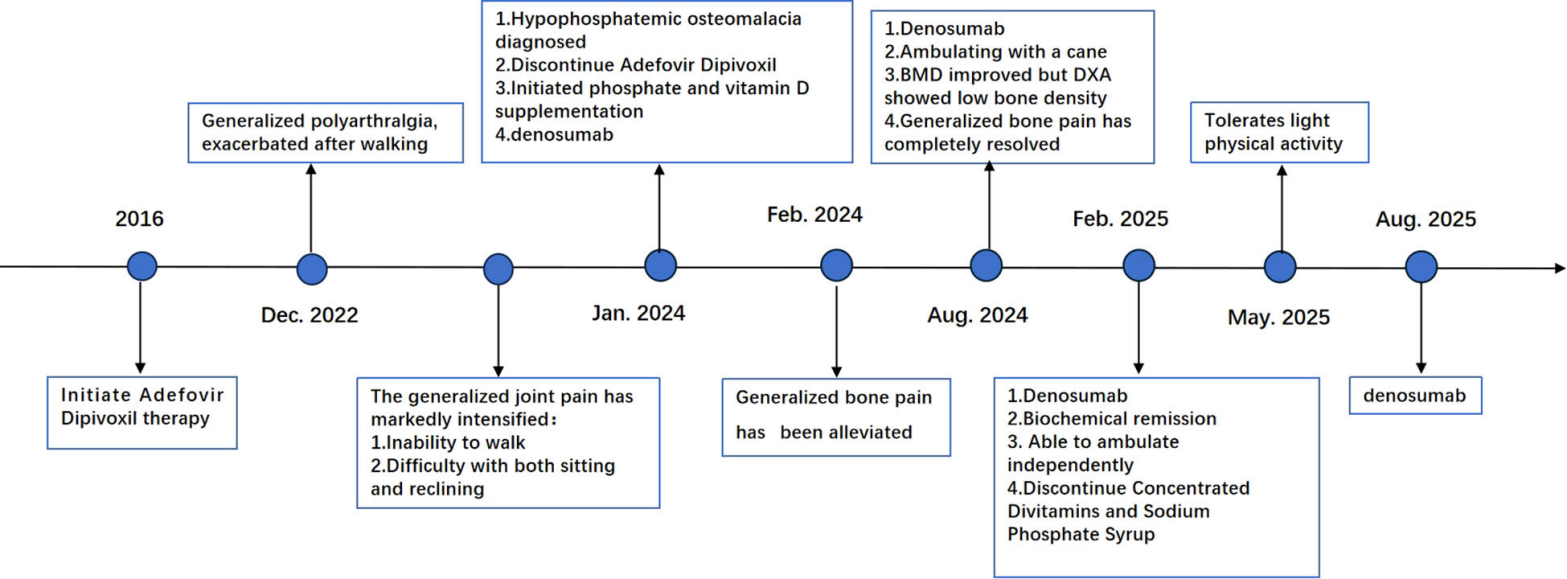
Patient diagnosis, management, and prognosis. DXA Dual-energy X-ray Absorptiometry ECTEmission computed tomograph.

Following confirmation of the diagnosis, ADV was discontinued. During hospitalization she received daily intravenous potassium phosphate solution (2 mL per ampule, containing 0.435 g potassium dihydrogen phosphate and 0.639 g dipotassium hydrogen phosphate) together with other supportive care. Following discharge, the patient was maintained on a regimen of fortified phosphorus syrup (10 mL, TID) providing 0.0162 g of elemental phosphorus daily, along with calcium and vitamin D supplementation. Denosumab (60 mg) was administered subcutaneously every six months (**Figure 3**). At the one-month follow-up after discharge, the patient’s generalized bone pain had improved. By six months, the pain had completely resolved. Seven months post-discharge, she was able to ambulate with a cane. At this time, her serum phosphorus levels had normalized, and her bone mineral density (BMD) showed significant improvement, although DXA still showed low bone density below the reference range for age-matched women (**Table 2**). After osteomalacia was largely resolved, given the patient’s postmenopausal status and inherent risk factors for postmenopausal osteoporosis, we determined that primary osteoporosis coexisted. Therefore, anti-osteoporotic therapy was initiated in addition to ongoing calcium and vitamin D supplementation. The subsequent anti-osteoporosis regimen consisted of denosumab 60 mg every six months plus continuous calcium and vitamin D supplementation. One year after discharge her alkaline phosphatase, bone-turnover markers, and arterial blood-gas profile had all normalized, and she was ambulating independently. By May 2025 she was able to perform light physical activity.

**Table 2:** Dual-energy X-ray absorptiometry (DXA) report: T-score.

Sites for bone density	Jan.2024	Aug.2024
Total Lumbar Spine	-5.3	-3.2
Left Hip Femoral Neck	-4.1	-2.0
Total	-4.7	-3.5
Distal Third of the Right Forearm	-4.6	-4.5

## 3 Discussion

### 3.1 ADV and nephrotoxicity

ADV is a widely utilized first-line medication for chronic HBV. After oral intake, it is metabolized to adefovir, which is subsequently phosphorylated by cellular kinases into its active form, adefovir diphosphate. This compound inhibits HBV DNA reverse transcriptase through two primary mechanisms: competitive antagonism against the natural substrate deoxyadenosine triphosphate (dATP) and termination of DNA chain elongation (6). Earlier studies, largely based on short-term data, regarded ADV as carrying a low risk of nephrotoxicity (7). However, subsequent reports demonstrate that therapy extending beyond 2 years confers a significantly higher incidence of renal injury than shorter treatment durations (8). Two meta-analyses have identified long-term ADV therapy as a significant risk factor for renal impairment. Both analyses confirmed that the risk is positively correlated with treatment duration and exhibits a cumulative nature (9, 10). The nephrotoxicity of ADV primarily targets the proximal tubule. Its specific accumulation within proximal tubule cells results from disrupted uptake via the organic anion transporter 1 (OAT1) and impaired secretion by apical multidrug resistance protein (MRP) (11). Furthermore, adefovir diphosphate inhibits human mitochondrial DNA polymerase- (mtDNA polymerase-). This inhibition depletes mitochondrial DNA (mtDNA) levels, disrupts oxidative phosphorylation, and consequently depletes cellular ATP in tubular cells, ultimately inducing nephrotoxicity (9, 12). A meta-analysis has shown that, in monitoring adefovir-related renal impairment, glomerular filtration rate (GFR) is a more sensitive indicator than serum creatinine, which may facilitate earlier clinical detection of kidney injury. (9). Because the risk of renal injury is cumulative, GFR should be the primary surveillance parameter in patients on long-term therapy, the elderly, and those with hypertension, diabetes, or pre-existing renal impairment, with regular assessment to enable early detection 104 and intervention for renal dysfunction.

### 3.2 Fanconi syndrome and hypophosphatemic osteomalacia

Fanconi syndrome is a rare disorder of proximal tubular transport; impaired reabsorption produces renal tubular acidosis, hypophosphatemia, phosphaturia, renal glycosuria, aminoaciduria, hypokalemia, and hypocalcemia (13). The nephrotoxicity of ADV disrupts multiple transport systems in the proximal tubule. This impairment affects the Na+-K+-ATPase pump function, leading to defective reabsorption of phosphate, bicarbonate, glucose, and amino acids (12). Consequently, prolonged ADV therapy can precipitate Fanconi syndrome (4, 14). A study has reported that adefovir-associated Fanconi syndrome may also produce peripheral neuropathy, although the underlying mechanism remains unclear (13). In recent years, numerous cases of adefovir-related Fanconi syndrome leading to hypophosphatemic osteomalacia have been reported (15–17). Prolonged urinary phosphate loss causes persistent hypophosphatemia. This blocks hydroxyapatite deposition in bone matrix. Unmineralized osteoid builds up and leads to osteomalacia. The clinical presentation includes bone pain, pseudofractures, fractures, and periodontitis. In severe adult cases, thoracic skeletal deformities may occur (18). This patient presented clinically with generalized bone pain. Imaging showed lumbar compression fractures, a left femoral-neck fracture, and multiple pseudo-fractures. Labs revealed renal tubular acidosis, low serum phosphate, and high urinary phosphate loss. We attributed these findings to adefovir-related Fanconi syndrome causing hypophosphatemic osteomalacia. Hypophosphatemic osteomalacia has many causes. Even after diagnosing Fanconi syndrome from ADV, we must still rule out other possible conditions.

Bone-specific alkaline phosphatase (BAP) can separate acute or subacute hypophosphatemia from chronic hypophosphatemia (18). Fibroblast growth factor 23 (FGF-23) is the key regulator and diagnostic marker of chronic hypophosphatemia. Its level distinguishes FGF-23-mediated from non-FGF-23-mediated disorders and is essential for identifying the cause of hypophosphatemic osteomalacia (18). X-linked hypophosphatemic rickets (XLHR) is the most common inherited form of hypophosphatemic rickets. It results from inactivating mutations in the PHEX gene (phosphate-regulating gene with homologies to endopeptidases on the X chromosome) (19). Current research identifies a key mechanism in XLHR. It involves significant upregulation of FGF23 transcription and synthesis in bone. This leads to elevated or high-normal serum FGF23 levels in patients. This pattern is a critical distinction from drug-induced injury, which typically shows low FGF23 levels (20). Tumor-induced osteomalacia (TIO) is a rare paraneoplastic syndrome caused by bone or soft-tissue tumors. These tumors are phosphaturic mesenchymal tumors (PMTs) that autonomously overproduce FGF23. Excess FGF23 blocks renal phosphate reabsorption and impairs active vitamin D synthesis, leading to acquired hypophosphatemic osteomalacia (19, 21). Locating the tumor is the main challenge in managing TIO. These tumors are often small and hard to find. Traditional imaging methods frequently miss them (21). Today, somatostatin-receptor functional imaging with Ga-DOTATATE PET/CT is the first-choice tool. It exploits the high somatostatin-receptor density on PMTs and offers whole-body screening with high sensitivity and specificity (22). Measuring serum FGF23 can effectively distinguish between drug-induced kidney damage and FGF23-mediated hypophosphatemia. This helps identify the cause of hypophosphatemic osteomalacia. However, FGF23 testing is not yet widely available in most hospitals and laboratories.

### 3.3 Hypophosphatemic osteomalacia versus primary osteoporosis

In practice, hypophosphatemic osteomalacia is often mistaken for osteoporosis because both cause bone pain and fractures. Their core pathology, clinical pattern, and lab values are clearly different. Osteoporosis is a loss of micro-architecture that lowers BMD and raises fracture risk. Osteomalacia is a primary failure of matrix mineralization that lowers mineral content. It causes widespread bone pain and typical proximal-muscle weakness. Osteomalacia shows persistent low blood phosphorus, high urine phosphorus, and very high alkaline phosphatase. In contrast, these levels are mostly normal in primary osteoporosis (23). Bone turnover markers help distinguish hypophosphatemic osteomalacia from osteoporosis. Osteomalacia often shows a large increase in the bone formation marker P1NP. It also shows a rise in the bone resorption marker CTX. In contrast, bone turnover levels vary greatly in osteoporosis (24).

This case is special because it has two diseases at the same time. The patient is a postmenopausal woman. Her age and hormone levels put her at high risk for primary osteoporosis. Her chronic HBV infection is another important risk factor. Chronic liver disease can cause hepatic osteodystrophy through several pathways. Its main features are reduced bone density and worsened bone structure (25). Studies show that about 70% of patients with chronic liver disease develop osteoporosis and face a high fracture risk (26). A Korean cohort study did not show a clear link (27).

However, a Mendelian randomization study in East Asians found a causal relationship between chronic HBV and osteoporosis (25). This supports the view that chronic HBV is an important co-factor for osteoporosis in this patient. Imaging showed very low bone density in this patient. However, this result needs careful analysis. Osteomalacia involves a buildup of unmineralized osteoid. DXA scans cannot tell the difference between fully mineralized bone and this osteoid. This leads to an underestimation of bone density, creating a “pseudo-low density” reading. Considering the patient’s profile, her long-term ADV use induced Fanconi syndrome, which subsequently led to hypophosphatemic osteomalacia. Concurrently, her postmenopausal status and chronic HBVhistory established a pathological basis for the development of osteoporosis. Therefore, she did not have a single bone disease. Decreased bone mass and impaired mineralization were both present. The combination of these two mechanisms explains her persistent bone pain, multiple fractures, and extremely low BMD that neither process could account for alone.

Few reports describe adefovir-induced hypophosphatemic osteomalacia together with osteoporosis. Correct diagnosis and proper treatment sequence are essential. Screen at once if any unclear bone pain, muscle weakness, or fracture appears in patients who have taken ADV for more than two years, especially postmenopausal women and other high-risk groups (8). The screening panel includes serum phosphorus, calcium, alkaline phosphatase, renal function, and urinalysis. If results suggest hypophosphatemia and renal tubular dysfunction, further testing with 24-hour urinary phosphorus, parathyroid hormone, and vitamin D levels is required to confirm the diagnosis and fully assess mineral metabolism status.

The key treatment is to immediately stop the kidney-toxic drug ADV. Patients should then switch to alternative antiviral agents, such as entecavir, which are associated with reduced nephrotoxicity and a decreased risk of Fanconi syndrome. (15). After stopping the harmful drug, the next key step is to correct the low phosphorus levels and mineralization problems. This is crucial for relieving symptoms and reversing the bone disease. According to Chinese guidelines, the standard treatment includes neutral phosphate salts. These are used together with active vitamin D or similar medicines (5). However, neutral phosphate buffer must be prepared by the user. This makes it less convenient for clinical use. For this patient, we first used intravenous compound potassium phosphate in the hospital. After discharge, we switched to an oral concentrated phosphorus syrup for long-term use. With this treatment, her blood phosphorus levels returned to normal. Her stubborn bone pain also improved significantly.

For anti-resorption drugs, current evidence shows a clear rule. Bisphosphonates should not be used in active osteomalacia. This is because they can block bone mineralization (15). Research on denosumab shows mixed results. Some reports indicate a risk in patients with Fanconi syndrome-related osteomalacia. Denosumab may cause severe low calcium or low phosphorus levels. It can even worsen bone pain (28, 29). Other studies show that after mineral deficits are fully corrected, denosumab given to patients with continued high bone turnover can relieve pain and lower turnover markers (30, 31). This matches what we saw in our case. Our patient’s bone pain disappeared after six months. Her bone turnover markers also returned to normal. This indicates that denosumab can be a treatment option for such patients, provided that adequate vitamin D and phosphate supplementation is ensured. Close monitoring of serum calcium levels is required during therapy. This field still faces many unresolved questions. Future studies should focus on the efficacy and safety of different anti osteoporosis agents, especially anabolic drugs, in patients with this dual pathology in order to build evidence based, individualized treatment protocols.

## 4 Conclusion

Adefovir-induced hypophosphatemic osteomalacia plus osteoporosis is a challenging dual disorder. The drug’s proximal-tubule toxicity produces Fanconi syndrome, raising urinary phosphate loss and lowering serum phosphate, which leads to osteomalacia. At the same time, the patient’s underlying postmenopausal status and chronic liver disease created a high-risk background for osteoporosis. The key steps are to stop adefovir and switch to a non-nephrotoxic antiviral, promptly correct hypophosphatemia and the mineralization defect, and only after mineral metabolism is stable, carefully decide whether anti-osteoporosis therapy is needed. In patients on long-term ADV who develop skeletal symptoms, clinicians should systematically screen tubular function to avoid missed diagnosis and shift management from symptomatic care to cause-directed therapy. Future research must target this unique comorbid group. It should test the efficacy and safety of different anti-osteoporosis drugs to design individualized sequential regimens, and it should develop biomarkers and risk models that identify high-risk patients early.

## Data Availability

The original contributions presented in the study are included in the article/supplementary material, further inquiries can be directed to the corresponding author/s.
